# Recent Developments in Organic Radical Inclusion in MOFs and Radical MOFs

**DOI:** 10.1002/open.202500069

**Published:** 2025-07-01

**Authors:** Christophe Adrien Ndamyabera, Henrietta Wakuna Langmi

**Affiliations:** ^1^ Department of chemistry University of Pretoria Private Bag X20 Hatfield 0028 South Africa

**Keywords:** host‐guest chemistries, inclusion MOFs, organic radicals, porosities, radical MOFs

## Abstract

Organic radicals are attractive materials due to their structures which contain unpaired electrons susceptible to charge transfer upon excitation. They possess potential properties such as optical properties, magnetism, and electrical conductivity. However, they are usually unstable impeding their further advancement and application. Organic radicals can serve as guest molecules in porous solids specifically metal–organic frameworks (MOFs) thereby gaining stability. MOFs are of interest due to their potential properties including large surface area and high adsorption capacity. Apart from their incorporation to form organic radical inclusion MOF hybrids, organic radicals can act as ligands in MOFs to yield radical MOFs. These hybrids and radical MOFs often exhibit enhanced properties such as improved catalytic, magnetic, optical, and sensing properties which make them promising for industrial applications. Herein, organic radical inclusion in MOFs and radical MOFs are reviewed. A brief background on organic radicals is presented. Different methods of integrating organic radicals (guests) in channels of MOFs (hosts) and the resulting changes in the physicochemical properties are documented. Furthermore, the use of organic radicals as ligands in the synthesis of radical MOFs is discussed as an alternative to organic radical–MOF inclusion compounds, and the ensuing physicochemical properties are highlighted.

## Graphical abstract

Inclusion of organic radicals in porous materials offers an opportunity to tailor properties that include stability of radicals, conductivity, sensing, and catalysis. Similarly, radical metal‐organic frameworks (MOFs) are synthesised to enhance or create such properties. This review provides details on methods of organic radical inclusion in MOFs, synthesis of radical MOFs, and potential properties.

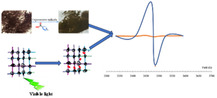

## 1 Introduction

The adsorption of guest molecules in channels or cavities of metal–organic frameworks (MOFs) is controlled by supramolecular interactions. Crystal structures of some MOFs have been reported to undergo transformation after adsorption or inclusion of guest compounds.^[^ ^1^ ^]^ In some cases, there have been observed reversible structural changes upon adsorption‐desorption.^[^ ^2^, ^3^ ^]^ Such MOFs are regarded as having flexible frameworks, while other frameworks considered as rigid remain intact upon adsorption‐desorption. However, the collapse of some structures has also been reported after sorption due to strong host‐guest interactions.^[^ ^4^ ^]^ MOFs are capable of acting as hosts for species such as organic radicals that may be highly unstable.

Organic radicals are molecules containing mainly atoms such as H, C, O, N, and S with an electronic configuration that makes them very reactive.^[^ ^5^, ^6^ ^]^ These molecules contain a group of atoms (radicals) with an unpaired electron occupying the highest molecular orbital. This facilitates reactions such as hydrogen abstraction, dimerization or recombination. Their reactivity allows them to coordinate with metals, forming a new class of compounds.^[^ ^7^, ^8^, ^9^ ^]^ It also allows incorporation into porous materials such as zeolites, activated carbon, and MOFs^[^ ^10^, ^11^, ^12^ ^]^ resulting in new materials with new potential applications.

Five‐membered 7π thiazyl radicals, S_3_N_2_^•+^ or six‐membered ring [S_3_N_3_]^•^ synthesized in 1880 can be considered as a starting point to synthesize some aromatic or anti‐aromatic organic radicals.^[^ ^6^, ^13^ ^]^ In 1991, Cordes et al. were then inspired to develop dithia‐ and diselenadiazolyl radicals as novel materials with properties for molecular semiconductor applications.^[^ ^6^, ^14^ ^]^ Organic radicals with dithiadiazolyl (DTDA) and dithiazolyl (DTA) rings were used as building blocks suitable for the preparation of organic conductors and magnets.^[^ ^6^ ^]^ It has been shown that electronic properties of materials prepared from these radicals respond very well to slight changes in solid‐state structure, with molecular conductors, magnets, as well as bistable materials all constructed from the same category of inorganic heterocycles. A good aspect of these materials is that they allow modulation of the properties.^[^ ^6^ ^]^ Hence, engineering of new materials for better performance of electron transport is feasible. Stable metal‐free organic radicals were initially synthesized via a combination of *N*‐(4‐methoxyphenyl)iminophosphorane and diphenylnitrylimine in 1969. The products are reported with very low yield (as low as 5.5%) due to the starting materials used, hindering their scalability and further investigation. In this regard, aza‐Wittig‐mediated synthesis was used to achieve moderate to good yields with a short time of synthesis.^[^ ^15^ ^]^ There are also nitroxide radicals known for their use in cross‐coupling reactions, which attract attention for their synthesis. They can establish weak N—O···X (X: halogen) bonding which is important in sensing and catalytic reactions.^[^ ^16^ ^]^ Oxime radicals are also stable depending on their substituents.^[^ ^17^ ^]^ They are reported for their applications such as catalysis, production of polymers, energy storage, and magnetic materials design. Furthermore, peracetic acid (PAA), has shown its potential as an alternative disinfectant, and its advanced oxidation processes (AOPs) could be useful for degrading pollutants.^[^ ^18^ ^]^ It was observed that PAA first undergoes a redox process vis‐à‐vis Co^II^/Co^III^ to obtain the active form for pollutant degradation. Owing to their unpaired electron, organic radicals are typically very reactive. However, the combination of pi‐delocalization (thermodynamic stabilization) and steric protection (kinetic stabilization) can lead to robust radicals which are air and moisture stable. Importantly, they are susceptible to performing a range of reactions or transformations.^[^ ^18^ ^]^ Organic radicals are prepared by different methods, and some are even difficult to synthesize or cannot be isolated as they decay extremely rapidly.^[^ ^19^ ^]^ However, persistent or stable radicals can be synthesized due to stabilization effects which prevent dimerization and other reactions that would otherwise destabilize the organic radicals.^[^ ^20^, ^21^ ^]^ These radicals include nitrogen‐centered amidyl, hydrazonyl, and imidyl radicals, Blatter radicals, nitroxides,^[^ ^20^, ^21^ ^]^ perchlorotriphenyl tricarboxylic acid radicals,^[^ ^22^ ^]^ 2,3′‐biimidazo[1,2‐α]pyridin‐2′‐one,^[^ ^23^ ^]^ and dithiadiazolyl^[^ ^11^, ^20^ ^]^ radicals. Considering the properties of organic radicals for potential applications, porous materials can be used to improve the feasibility via inclusion in solid state. Moreover, the properties of porous coordination polymers where organic radicals can serve as linkers would be beneficial.

In this review, we have considered organic radicals as guest molecules in MOFs (organic radical inclusion MOFs) and as ligands in MOFs (radical MOFs). We have reviewed different inclusion methods used so far to incorporate organic radicals as guests in MOFs. In addition, strategies for generating radical MOFs are discussed. Furthermore, features and reactivity of the resulting hybrids of inclusion as well as radical MOFs are examined. Finally, the influence of the inclusion of organic radical moieties in MOFs on physical and chemical properties is presented. Various radicals are discussed but those possessing S or N atom are of interest because they may offer opportunity for additional supramolecular interactions. Although this review highlights the work done by Faust and D’Alessandro,^[^ ^10^ ^]^ more importantly, it also provides new information and recent research work on radical inclusion in MOFs which are not available in the above review. More details on stabilization of some radicals once introduced into MOFs are discussed. Radical MOFs prepared by direct synthesis, that is, in situ are presented here compared to the previous work. In addition, more redox agents for the creation of radical MOFs are presented. Reactions that include catalysis in radicals@MOF are detailed, and further work on radical MOF applications such as photothermal conversion in these materials are presented. This work also discusses how organic radicals can be used in sensing not only new molecules but also the level of mechanical shock in the frameworks.

## 2 Features of Organic Radicals and MOFs

### 2.1 Features of Organic Radicals

In an organic radical, the unpaired electron is located in an antibonding π* or σ*‐orbital offering a highly reactive nature.^[^ ^18^ ^]^ It has been suggested that the stability of the radical is enhanced by the delocalization of electrons on several atoms that stabilize the π* orbital (SOMO).^[^ ^5^ ^]^ Organic radicals can act as a ligand, subsequently playing an important role to achieve ferrimagnetic chains (magnetic coupling degrees).^[^ ^24^ ^]^ Some organic radicals are characterized by appreciable solubility, which is an important factor for stability in electrochemistry.^[^ ^25^ ^]^ Organic radicals possess energy gap defined by the highest occupied molecular orbital (HOMO) and the lowest unoccupied molecular orbital (LUMO). When the energy gap of HOMO–LUMO is large the material is an insulator. This can be reduced by removal of electrons from the HOMO or adding electrons to LUMO to allow conductivity.^[^ ^26^ ^]^ Moreover, organic radicals are able to establish supramolecular interactions, which is an important feature in solid‐state reactions for potential applications.^[^ ^5^, ^27^ ^]^ These interactions include π···π, Cl···Cl, H···π, electrostatic interaction, and hydrogen bonding. The classification of organic radicals has been reported in a previous review work by Faust and D’Alessandro.^[^ ^10^ ^]^

### 2.2 MOFs

Porous materials such as activated carbon, zeolites, and MOFs play an important role in solid‐state reactions.^[^ ^12^, ^28^, ^29^, ^30^ ^]^ Their porous structures allow the inclusion of small molecules via the influence of weak interactions and/or coordination bonding.^[^ ^31^ ^]^ Practical application of these materials, most often, capitalies on the reversible accommodation of guests within the host material. MOFs are formed by metal centers bridged by organic linkers, resulting in polymer networks.^[^ ^2^, ^32^ ^]^ Hence, they are 3D or 2D structures that contain channels or pores.^[^ ^32^, ^33^ ^]^ The latter have different sizes that play a part in the magnitude of surface area and the size of guest molecules that can be accommodated.^[^ ^4^, ^32^ ^]^ However, some of them can respond to the stimuli of guest molecules with flexibility to accommodate a larger size of guest molecules depending on the MOF.^[^ ^3^, ^34^, ^35^ ^]^

MOFs are attractive due to their potential properties that include high surface area, porosity with a possibility to withstand high heat and chemical stimuli, high adsorption capacity, and tunability.^[^ ^32^, ^36^, ^37^, ^38^, ^39^ ^]^ The synthesis of MOFs offers opportunities for designing desired frameworks for potential applications with well‐defined characteristics. MOFs also allow functionalization which can be performed via in situ or postsynthetic approach. These materials are reported in different applications such as catalysis, drug delivery, sensing, and gas storage.^[^ ^29^, ^40^, ^41^, ^42^ ^]^ Moreover, they are recoverable materials for repetitive applications, which is an important property for industrial applications.^[^ ^3^, ^43^ ^]^

It is worth noting that studies of host‐guest chemistry in MOFs are at the cutting edge of their effectiveness to meet industrial applications. Postsynthetic or in situ transformation is among strategies adopted where the crystal structure of the original MOF is maintained.^[^ ^44^, ^45^ ^]^ In this regard, functionalization of MOFs that imparts better adsorption has been applied.^[^ ^46^ ^]^ Furthermore, properties of MOFs such as magnetic properties, can be created or improved via the inclusion of organic radicals that are already magnetic.^[^ ^11^ ^]^ The change in these properties is due to supramolecular interactions established between MOFs and organic radicals.^[^ ^10^, ^31^ ^]^ Generally, once the radicals interact with the channels of porous material, it results in an increase in the total number of spin centers present in the sample and influences the properties.^[^ ^11^ ^]^ As both MOFs and organic radicals have extensive applications, their combined effect can play an important role in industrial applications.

## 3 Inclusion of Organic Radicals in MOFs and Synthesis of Radical MOFs

Inclusion of organic radicals in porous solid materials such as MOFs is important not only in preparation of new materials but also in improving the properties of both organic radicals and hybrids of MOF‐organic radicals.^[^ ^10^, ^47^ ^]^ The induced changes depend on the structure of pores or channels of the host and the chemical properties of both the organic radical and host that allow interactions.^[^ ^48^ ^]^ Thus, it is interesting to study potential characteristics due to the resulting changes. Once guest molecules such as radicals enter the channels of MOFs, they may establish either radical…host or radical…radical interactions and both types of interactions can be observed in the same system.^[^ ^32^, ^49^ ^]^ Therefore, this can affect the kinetic energy for the removal of these guest molecules^[^ ^32^, ^49^ ^]^ or affect responsiveness to external stimuli by the host. However, in some cases, organic radicals can play the role of a ligand through coordination reaction, while other MOFs or metal complexes can undergo postsynthetic modification by transforming a part of the linker into an organic radical.^[^ ^44^, ^50^, ^51^ ^]^ These offer a wide range of opportunities to generate radical MOFs with the potential to obtain targeted properties. Creating radical ligands in MOF networks in postsynthesis modification may be achievable to a certain percentage in the framework.^[^ ^52^ ^]^ Organic radicals discussed here are generally stable, and those formed in situ get some protection from the framework.

**Scheme 1:**
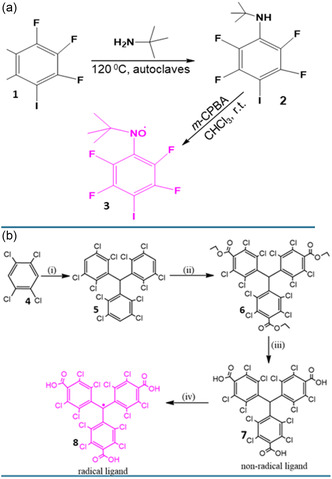
Formation process of radical ligands for their incorporation in MOFs as a) guest molecules. Adapted with permission.^[^ ^16^ ^]^ Copyright 2024, MDPI and b) linkers. Reproduced with permission.^[^ ^22^ ^]^ Copyright 2024, American Chemical Society.

We have selected two stable organic radicals to illustrate their corresponding synthesis in **Scheme** **1**. An example of the formation of nitroxide radicals, known to be useful guest molecules or decorated linkers in MOFs, is presented in Scheme 1a. The substitution of a fluorine atom in a polyfluorinated arene (**1**) by *tert*‐butylamine afforded the derived *N*‐*tert*‐butylaniline (**2**). This was followed by the oxidation of **2** with *meta*‐chloroperoxybenzoic acid (m–CPBA) that led to the formation of *tert*‐butylphenylnitroxide radicals (**3**).^[^ ^16^ ^]^ Another example is the synthesis of perchlorotriphenyl tricarboxylic acid radical ligands displayed in Scheme 1b.^[^ ^22^ ^]^ Tris(2,3,5,6‐tetrachlorophenyl)methane (**5**) was first synthesized from Friedel–Crafts alkylation of 1,2,4,5‐tetrachlorobenzene (**4**) with CHCl_3_ in the presence of AlCl_3_. The product was then treated with n‐BuLi, tetramethylethylenediamine, and ethyl chloroformate in anhydrous THF to form the perchlorotriphenylmethyl triester (**5**). The reaction of **5** with concentrated sulfuric acid formed a nonradical perchlorotriphenyl tricarboxylic acid (**6**), ligand. Finally, the nonradical ligand, **6,** was deprotonated using NaOH and subsequently oxidized by ***I***_**2**_ to afford radical perchlorotriphenyl tricarboxylic acid (**7**) at appreciable yield. The latter radical ligand was used in the synthesis of radical MOFs as described in Section 3.2.

Organic radicals can be incorporated into porous MOFs via different routes, such as gas phase diffusion,^[^ ^11^ ^]^ solution diffusion,^[^ ^48^ ^]^ or in situ synthesis^[^ ^7^, ^53^ ^]^ followed by a change in physicochemical properties. The incorporated organic radicals (guests) can subsequently be recovered from porous MOFs on heating or using solvents such as dichloromethane.^[^ ^11^ ^]^ Solvothermal or room‐temperature synthesis is also used to incorporate organic radicals in MOF networks.^[^ ^54^, ^55^, ^56^ ^]^ The use of heating, redox agents and irradiation are techniques used to create radical moieties in MOFs. Further details on the inclusion of organic radicals in MOFs and the preparation of radical MOFs are discussed in the next two sections.

### 3.1 Methods of Organic Radical Inclusion in MOFs

**Figure 1:**
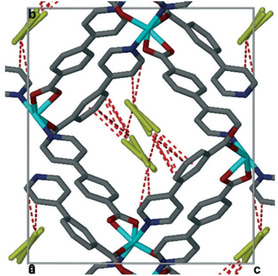
A typical example of channels of Co(34pba)(44pba) MOF easily accommodates iodine guest molecules. Adapted with permission.^[^ ^28^ ^]^ Copyright 2021, Royal Society of Chemistry.

Different methods of inclusion are performed depending on the nature of the host or the guest molecules. Thus, the pore size of MOFs and the polarity of organic radicals must be able to allow the flow of guest molecules and mutual guest‐host interactions.^[^ ^32^, ^53^, ^57^ ^]^ However, some linkers of the framework can also adopt libration motion during the adsorption process.^[^ ^58^ ^]^ This is because some porous materials may possess small pore sizes in which big molecules cannot be hosted. **Figure** **1** displays an example of pore size compared to guest molecules and guest‐host interactions. Small guest molecules will occupy the channels and establish supramolecular interactions easily, which will be different for a big size of guest molecules. Two guest molecules with different sizes hosted in the same MOF can be compared. [Co(34pba)(44pba)], where 44paba is (4–(4–pyridyl)benzoate and 34pba are 3‐(4‐pyridyl)benzoate ligands, was used to investigate adsorption of iodine molecules.^[^ ^28^ ^]^ It can be seen that the size of iodine molecules (in lime color) can easily fit in the window of the open channel of the [Co(34pba)(44pba)] MOF. This adsorption led to a ratio of host:guest of 1:0.8. While, when iodobenzene which has a bigger kinetic diameter was used as guest molecules, the host:guest ratio was 1:0.05. Additionally, using ammonia (small kinetic diameter) and 1–phenylethylamine (bigger kinetic diameter) for example, the ratio of MOF: ammonia was 1:4.6 while the one for MOF: 1‐phenylethylamine was 1:035.^[^ ^32^ ^]^ Hence, the adsorption of larger guest molecules can be low or even absent depending on the host flexibility.^[^ ^57^ ^]^

**Figure 2:**
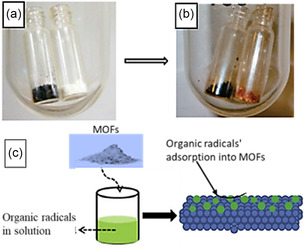
On the top, organic radicals and MOFs in a closed chamber a) before and b) after adsorption. Reprinted with permission.^[^ ^59^ ^]^ Copyright 2011, American Chemical Society, c) MOFs suspension in a solution containing organic radicals followed by adsorption. Adapted with permission.^[^ ^48^ ^]^ Copyright 2015, Elsevier Ltd.

It is evident that the inclusion of organic radicals in MOFs depends on their volatility or solubility. These properties will dictate the inclusion methods to access the channels of the frameworks. One approach involves the inclusion of organic radicals in MOFs by exposing MOFs to subliming organic radicals.^[^ ^59^ ^]^ Due to the volatility of some organic radicals, they are heated under vacuum and simultaneously exposing MOFs to the resulting vapors for inclusion to occur.^[^ ^11^ ^]^ After a certain time, the organic radicals sublime and diffuse into channels of MOFs. The second method is impregnation where organic radical compounds are first dissolved in a solution. Then, solid MOFs are added to the solution as a suspension for adsorption. The organic radicals are then taken up in pores/channels through adsorption.^[^ ^48^ ^]^ Both methods of radical inclusions can be depicted in **Figure** **2**.

**Figure 3:**
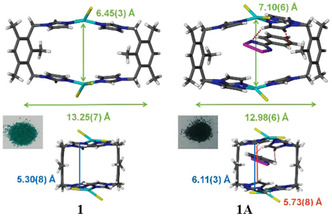
Change in pores size in the frameworks associated with MOF‐guest radical interactions in [Cu_2_(L_1_)_2_Cl_4_](top) and its related color change Reproduced with permission.^[^ ^11^ ^]^ Copyright 2017, Royal Society of Chemistry.

The inclusion can be characterized by a color change and even the change in texture of the solid material. **Figure** **3** shows that the inclusion of phenyl–1,2,3,5–dithiadiazolyl radical (PhCNSSN^•^) into porous metallocycle, green [Cu_2_(L_1_)_2_Cl_4_] where L_1_ = 1,3‐bis(imidazol‐1‐ylmethyl)‐2,4,6‐trimethylbenzene) named as **1** led to **1A**, a dark red material.^[^ ^11^ ^]^ From the left to the right of the figure, the guest molecules on the right side are held into the framework by hydrogen bonding and other weak interactions. Considering the top images, the vertical green arrow shows expansion while the horizontal one shows contraction. At the bottom of the images from the left to the right, the arrow shows the expansion of the centroids on the imidazole rings. This is referred to as single‐crystal‐to‐single‐crystal change due to host‐guest interactions.^[^ ^11^ ^]^ Thus, these changes in the frameworks are in this case, responsible for the color change.

### 3.2 Synthesis of Radical MOFs

MOFs are generally made of nonorganic radical ligands and offer porous/channel structures. They can allow the entering of guest molecules including organic radicals into their porous structures and form host‐guest interactions. Instead of being guest molecules, some organic radicals have been used to synthesize radical MOFs in situ. Postsynthetic reaction can also be performed where part of the organic linker of the MOF is transformed into a radical to achieve radical MOFs for intended application or study.^[^ ^1^, ^50^ ^]^ This is performed using external stimuli such as heat, IR, UV and visible radiation, and electron donor compounds.^[^ ^54^, ^60^, ^61^, ^62^, ^63^ ^]^

#### 3.2.1 Postsynthetic Radical Formation in MOFs

**Figure 4:**
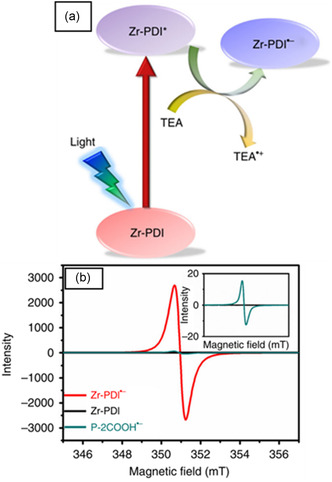
a) Photoinduced electron transfer process between TEA and Zr‐PDI, excited state Zr‐PDI* is reductively quenched by TEA to afford Zr‐PDI•‐. Reprinted with permission.^[^ ^53^ ^]^ Copyright 2019, Nature Communications. b) EPR spectrum of Zr‐PDI and Zr‐PDI^•−^ highlighting the enhancement of the EPR spectrum after radical creation. Reprinted with permission.^[^ ^53^ ^]^ Copyright 2019, Nature Communications.

**Figure** **4** exhibits a comparison between Zr‐PDI (PDI = perylenediimide), formed by N,N′‐di‐(4‐benzoic acid)‐1,2,6,7‐tetra‐chloroperylene‐3,4,9,10‐tetracarboxylic acid diimide (P–2COOH) ligand and Zr_6_(μ_3_‐O)_4_(μ_3_‐OH)_4_ clusters, before radical formation and its corresponding radical Zr‐PDI^•−^.^[^ ^53^ ^]^ The radical MOF was formed upon irradiation of Zr‐PDI after trapping organic amines such as triethylamine (TEA), ethylenediamine (EDA), and tripropylamine (TPA). Irradiation using blue light (455 nm) in the presence of amines induced the electron transfer from amines to the linker of the framework. The excited state of Zr‐PDI* is then reduced to gain a stable form. For example, TEA releases electron to the Zr‐PDI*. This results in anion radical MOF, Zr‐PDI^•−^ trapping amine radical cations (TEA^•+^) in channels, Figure 4a. Zr‐PDI^•−^ MOF showed high electron paramagnetic resonance (EPR) intensity while that of Zr‐PDI was null as it can be observed in Figure 4b. Its corresponding ligand in the form of a radical, P–2COOH^•−^ was also characterized by weak intensity (spectrum in green). This is because this free ligand is unstable and oxidizes fast, while the radical MOF can last at least a month. Heating Zr‐PDI^•−^ at 120 °C was characterized by the leak of some amines leading to 89% of residual Zr‐PDI^•−^. This implies that on the removal of guest molecules from the channels of the MOF, the radicals disappear. In this case, such radical MOFs can be categorized into organic radical inclusion MOFs or radical MOFs. On the other hand, it was noted that when amines with low boiling points such as diethylamine (DEA), isopropylamine (IPA), and trimethylamine (TMA) were treated with Zr‐PDI, the radical properties could not last longer. Their boiling points are 55, 44, and 3 °C, respectively, while those for TEA, EDA, and TPA are 90, 116, and 155 °C respectively.

**Scheme 2:**
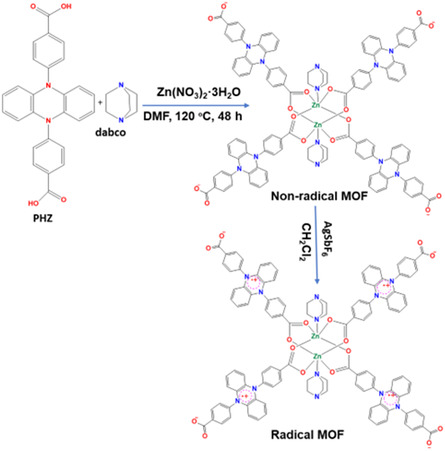
Illustration of the synthesis of a radical MOF via postsynthesis method using an oxidizing agent. Reproduced with permission.^[^ ^67^ ^]^ Copyright 2021, John Wiley and Sons.

Su et al. prepared MOFs using lanthanide nodes and redox‐active tetrathiafulvalene‐tetrabenzoate (TTFTB^4−^), which contains a neutral diamagnetic TTF moiety.^[^ ^50^ ^]^ The preparation was followed by either a solid‐solution electrochemical method or iodine (*I*_2_) oxidation to engineer the linker into a paramagnetic TTF^•+^ radical, facilitating a single‐crystal‐to‐single‐crystal transformation while maintaining the framework's structure intact.^[^ ^50^, ^64^ ^]^ In the case where *I*_2_ was used, a solid material MOF containing TTF was suspended in a cyclohexane solution of *I*_2_. The solid material became darker while purple *I*_2_ turned colorless. The darker color of the MOF implied the radical formation.^[^ ^64^ ^]^ This is confirmed by some characterization that includes EPR, where its resonance is at g ≈ 2. Moreover, the darker materials showed an additional absorption band around 750 nm, which was not observed for the same sample before *I*_2_ treatment. The formation of TTF^•+^ is made possible since TTF is a conjugated molecule rich in sulfur, making it easily oxidized to the radical form. Upon heating these radical MOFs prepared using iodine oxidation, iodine is released below 330 °C, while the framework remains stable up to 490 °C. The radical moieties could persist even after the release of iodine,^[^ ^65^, ^66^ ^]^ but this may be temporary due to potential exposure to air or moisture. A similar oxidizing agent was used wherein Zn_2_(PHZ)_2_(dabco)·4DMF (PHZ: 5,10‐di(4‐benzoic acid)‐5,10‐dihydrophenazine, dabco: triethylene diamine) was treated with AgSbF6 in dichloromethane, partially oxidizing it to a radical MOF.^[^ ^67^ ^]^ In the study, the PHZ and dabco coligand coordinated with Zn to form a MOF. Yellow crystals of the MOFs were treated with AgSbF_6_ using dichloromethane as solvent. The yellow MOF material then changed to dark green color, suggesting radical formation. It has been reported that PHZ derivatives easily undergo one‐electron oxidation to form colored radical cation species characterized by color change.^[^ ^67^ ^]^ They are highly π–conjugated and contain donor atoms that facilitate electron transfer. Therefore, the MOF underwent oxidation through PHZ, leading to a radical linker as presented in **Scheme** **2**. EPR spectra before and after oxidation by AgSbF_6_ were recorded. A very intensive EPR signal was observed for the solid after oxidation, proving the formation of radical species. This linker could remain stable for up to a month when kept at ambient temperature, demonstrating its resistance to reactions with oxygen and water.

**Figure 5:**
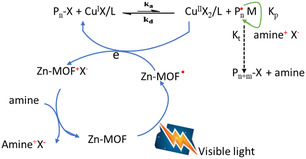
Illustration of MOF induced radical by irradiation. Adapted from permission.^[^ ^102^ ^]^ Copyright 2016, Green Chemistry.

UV–vis irradiations are also used for the formation of radical linker in MOFs. This was observed in Zn–MOF solvothermally prepared using terephthalic acid and [9,10‐bis(4‐pyridylethynyl)‐anthracene] (*L*_1_), where the latter ligand is a photoactive anthracene‐derived bipyridine.^[^ ^68^ ^]^ Once Zn–MOF is irradiated, a radical Zn–MOF is generated. According to **Figure** **5**, the radical MOF can then be used in catalytic reactions by transferring electrons to a copper catalyst for a reduction process. Similarly, the preparation of [Zr_6_(μ‐O)_4_(μ‐OH)_4_(C_6_H_5_COO)_*x*_(OOCCH_3_)_4−*x*_ (BBI)_2_] radical MOF, where BBI is 4,4′,4″,4‴‐(1,4‐phenylenebis(1H‐imidazole‐2,4,5‐triyl))‐tetrabenzoate, was achieved using UV light irradiation.^[^ ^60^ ^]^ When the temperature of this radical MOF increased from 100 to 225 K, isolated radicals were modulated into radical π‐dimers. This was possible due to the reduced distance between adjacent linkers which also depends on the guest molecules in the channels. When placed in the dark, the radical species in these materials decay as a reversible process.

**Figure 6:**
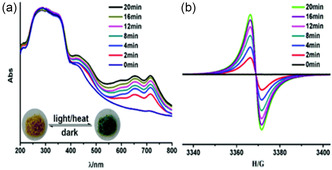
Gradual increase in intensity of a) UV–vis spectra and b) EPR signals associated with irradiation exposure time of [Zn(ipbp)(H_2_O)]·NO_3_·H_2_O. Reprinted with permission.^[^ ^62^ ^]^ Copyright 2016, Inorganic Chemistry Frontiers.

The generation of radical species is evidenced by the EPR spectrum after irradiation, which may sometimes be accompanied by a color change.^[^ ^60^, ^69^ ^]^ This property was observed in viologen‐based MOFs containing donor‐atoms such as oxygen that can transfer an electron to acceptor atoms such as nitrogen through intraligands or interligands process. Therefore, the presence of donor‐atoms and acceptor–atoms systems in the frameworks is necessary for electrons upon stimuli. However, the EPR signals may decay after sometimes when the radical MOF is placed in the dark and concomitantly the color change may be reversed; however, this varies from compound to compound.^[^ ^62^ ^]^ Reversible photoswitchings without a loss of color were recorded in such compounds.^[^ ^62^, ^63^ ^]^ The exposure time for irradiation is very crucial, as the EPR signal increases with longer exposure times^[^ ^62^ ^]^ to achieve a certain transformation of the ligand to the radical ligand.^[^ ^67^ ^]^ **Figure** **6** illustrates the effect of irradiation time on generating radical MOFs. This was observed in [Zn(ipbp)(H_2_O)]·NO_3_·H_2_O where ipbp is 1‐(3,5‐dicarboxyphenyl)‐4,4′‐bipyridinium bromide, where there is a gradual increase in intensity of UV/Vis spectra and EPR signals from the original, depending on irradiation time.^[^ ^62^ ^]^

Organic radical ligands were designed and used to construct MOFs with improved stability and efficiency of application. Herein, a radical MOF, [Zr_6_(μ_3_‐OH)_8_(OH)_8_](L‐OH)_2_ (L‐OH: 2,2′‐dihydroxy‐1,1′‐binaphthyl‐3,3′,6,6′‐tetrakis(4‐benzoic acid)) was synthesized via solvothermal synthesis using ZrCl_4_, L‐OH, and formic acid in DMF at 120 °C.^[^ ^61^ ^]^ The presence of a hydroxyl group of the ligand in the framework offers the formation of a radical once irradiated. Irradiation stimulates the hydroxyl group to perform an intramolecular electron transfer along with excited state intramolecular proton transfer, leading to oxyl radical. After a set of experiments, the resulting radical MOF was characterized by its advanced catalytic capacity with stability towards selective oxidation of sulfides with good recyclability.

**Figure 7:**
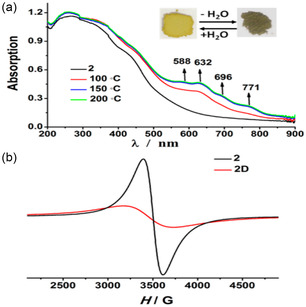
a) UV–vis bands of [Mn_2_(L)(N_3_)_2_(H_2_O)_2_]·3H_2_O showing new band due to radical formation upon heating and removal of water molecules, b) EPR spectra confirming radical formation. Reprinted with permission.^[^ ^1^ ^]^ Copyright 2017, American Chemical Society.

It is worth noting that postsynthetic generation of radical MOFs is performed where the position of radical transformation is on organic ligands and not on metal nodes. The creation of radical MOFs from nonradical MOFs was also reported, where guest solvents stimulated radical properties in MOFs. It may look like these MOFs are converted into radical MOFs via a simple process of photoirradiation, however, this depends on the nature of the ligands.^[^ ^62^, ^68^ ^]^ The presence and the closeness of donor‐acceptor moiety in a ligand play a role in electron transfer that allows radical formation. The capacity of a ligand to induce radical formation in a MOF varies depending on the specific MOF system, attributable to the influence of the metal center and the framework's topology. The metal center governs the electronic and redox properties, which modulate electron‐transfer processes, while the framework's topology dictates the spatial arrangement of metal nodes and linkers, thereby influencing the efficiency of electron‐transfer pathways within the ligand upon photoirradiation.^[^ ^1^ ^]^ Consequently, these factors collectively determine the ligand's ability to facilitate radical generation in a given MOF. Photoinduced irradiation on [Co_3_(L)(N_3_)_4_] (L^2−^: *N,N*′‐bis(3,5‐dicarboxylatobenzyl)‐4,4′‐bipyridinium) triggers azide−carboxylates moieties to transfer an electron to viologen and this was characterized by reversible photochromism leading to the formation of radical MOF, **1P**. However, upon photoirradiation of [Mn_2_(L)(N_3_)_2_(H_2_O)_2_]·3H_2_O (**2**) as in **1**, there was no radical formation. This is because the coordination environment of Co centers in **1** allows the interactions between electron‐poor viologen units and electron‐rich sites (either carboxylate or azide nitrogen) which is not the case in **2**. On the other hand, [Mn_2_(L)(N_3_)_2_(H_2_O)_2_]·3H_2_O (**2**) was transformed into a radical MOF upon removal of water molecules by heating and generating **2D** as a radical phase. **Figure** **7**a shows the UV absorption bands whereby new absorption bands indicated the formation of radicals. Before heating, compound **2** had a strong absorption in the region between 230 and 450 nm and no band after 450 nm (Figure 7a). After heating at 150 °C with full removal of water, new bands appeared at 588, 632, 696, and 771 nm in **2D**. These bands confirm the formation of radical species as evidenced by EPR spectrum in Figure 7b. X‐ray photoelectron (XPS) spectroscopy was used to identify the acceptor and donor atoms by considering the shifted peaks and found that azide groups may have transferred electrons to bipyridinium. To confirm that water removal induced electron transfer, rehydration was carried out, resulting in quenching of radical species. In this regard, it is evident that the coordination and conformation underwent changes that favor electron donors and acceptors. This could then allow the electron transfer from azide−carboxylate to bipyridinium. The compound **2D** was kept in dry air for a month without any change in EPR spectra at ambient temperature, while **1P** was only kept for several days in air before turning to **1**. However, **1P** could be stored for two weeks under nitrogen atmosphere. Reversible change in solid state of both **1** and **2** could be performed several times without structure deterioration.^[^ ^1^ ^]^

For diamagnetic MOFs without the possession of any radical moieties in the framework, they may have relatively null EPR intensity in a magnetic field. EPR is a very sensitive technique for the characterization of stable radicals. The intensity of the EPR spectrum increases significantly in intensity during inclusion and/or radical generation in MOFs. Postsynthetic radical formation in MOFs can also include perylenediimide‐based MOFs that trap electron donors such as amine vapors during solvothermal synthesis.^[^ ^53^ ^]^ After then, amine vapors create radical anions in the frameworks through photoinduced electron transfer.

#### 3.2.2 Direct Radical Formation in MOF during Synthesis

**Scheme 3:**
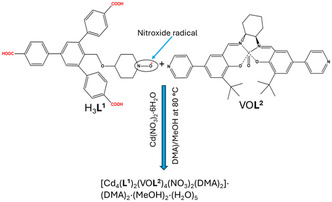
Direct synthesis of a radical MOF using mixed organic radical and nonradical linkers. Adapted with permission.^[^ ^7^ ^]^ Copyright 2018, American Chemical Society.

Other organic radicals are used in synthesis to coordinate metal cations for preparing ferromagnetic compounds characterized by intramolecular exchange interactions between metal cations and radical spins.^[^ ^16^ ^]^ (2,2,6,6‐tetramethyl‐ piperidin‐1‐yl)oxyl (TEMPO)‐substituted tricarboxylate (**L** ^**1**^) and enantiopure VO(salen)‐derived dipyridine ligand radicals (**VOL** ^**2**^) were used to construct a radical MOF based on a Cd metal center (**Scheme** **3**).^[^ ^7^ ^]^ **L** ^**1**^ is already a radical ligand that contributed to the formation of the radical MOF. The synthesis was conducted via a solvothermal method to yield crystal MOFs. The resulting radical MOF contained open channels with dimensions of ≈1.73 × 0.86 nm^2^, which efficiently served for catalytic processes. Structure elucidation finds that the radical moieties consistently decorate the pores.

Moreover, in situ radical MOFs synthesis has been reported to depend on the arrangement of the frameworks, the type of ligand, and the electron‐donor used. Naphthalenediimides (NDIs) ligands and Ca^2+^ metal center were used to synthesize radical DGIST‐7, [Ca_4_(μ_4_‐O)(L_2_^•−^)_2(1–*n*)_(L_2_^2−^)_2*n*_ (DMA^+^)_4‐2 *n*_(DMA^+^)_4 *n*_] (DMA^+^, dimethylammonium; 0 < *n* < 1, L^2−^ = N,N′‐bis(3‐hydroxybenzoate)‐1,4,5,8‐naphthale‐nediimide) using dimethylformamide solvent (DMF).^[^ ^54^ ^]^ The reaction was performed at 150 °C where DMF decomposed to release an electron to NDI for reduction, forming a radical, NDI^•−^. The characterization showed that the framework consists of the centroid‐to‐centroid distance between NDI planes of only 3.207 Å. This short distance allows π‐stacking interactions between adjacent linkers with electron delocalization, to generate a stable radical MOF.^[^ ^54^ ^]^ The radical form of DGIST‐7 could then be quenched by immersion into DMF. The resulting phase was treated with ethanol as a solvent exchange to form Oxi‐DGIST‐7. Interestingly, the compound formed radical MOFs upon exposure to heat (150 °C), IR, UV, visible light, and ammonia.^[^ ^54^ ^]^ However, EPR intensity was only high with the heating process. The coordination of some organic radicals to metals can successfully be achieved through ligand exchange.^[^ ^70^, ^71^ ^]^ Hence, 1D, 2D, and 3D structures were reported.^[^ ^5^, ^24^ ^]^ There are also organic radical ligands doped during the synthesis of MOFs to allow the magnetostructural functionalities.^[^ ^72^, ^73^ ^]^ This method was employed to achieve controlled magnetic interactions. It was noted that the doping must also be controlled because a high doping load results in structural rearrangement and pore defects, with even the loss of porosity.^[^ ^72^ ^]^ However, the structural rearrangement can be overturned or controlled through the control of temperature.

**Figure 8:**
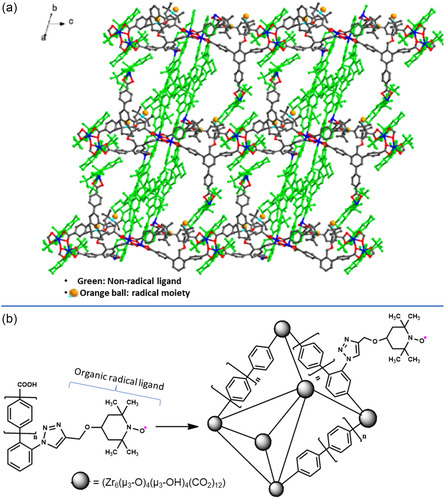
a) MOF decorated by organic radicals (in orange balls). Reprinted with permission.^[^ ^7^ ^]^ Copyright 2018, American Chemical Society, b) UiO‐67 decorated by TEMPO (radical in pink dots). Reprinted with permission.^[^ ^74^ ^]^ Copyright 2017, American Chemical Society.

Decorating MOFs with organic radicals can be done by cocrystallising two organic ligands where one is an organic radical, with a metal center. Organic ligand coordinates with metal center forming networks. These networks are in return interlinked by an organic ligand containing radical moieties.^[^ ^7^ ^]^ As an example, **Figure** **8**a shows enantiopure VO(salen)‐derived dipyridine ligands (bright green) coordinated to Cd metal centers while the rest colorful linker is a (2,2,6,6‐tetramethylpiperidin‐1‐yl)oxyl (TEMPO)‐substituted tricarboxylate. The latter (radical ligand) contains oxygen atoms of TEMPO, shown as light‐orange balls which decorate the channels of the MOFs.

Similarly, terephthalate‐TEMPO (BDC‐TEMPO) and biphenyldicarboxylate‐TEMPO (BPDC‐TEMPO) in different percentages were mixed with BDC and BPDC, respectively to prepare UiO–67–TEMPO or UiO‐66‐TEMPO (Figure 8b) via solvothermal conditions.^[^ ^74^ ^]^ Different ratios of unsubstituted linkers and substituted TEMPO linkers were used in the synthesis to monitor their effect on accessible pore volume. There was a challenge in obtaining MOFs with good stability against the temperature. The rectification was done by using HCl as a modulator in the synthesis. Even though this procedure resulted in defective materials, there was the presence of accessible pore volumes and surface areas higher than those of the parent MOFs.^[^ ^74^ ^]^ Organic radical ligands can also coordinate with the metal centers, forming a framework. In another study, radical perchlorotriphenyl tricarboxylic acid mixed with its nonradical form constructed a framework with Zn(II) metal centers.^[^ ^22^ ^]^ The two forms of the ligand and the *d* ^[^ ^10^ ^]^ closed‐shell configuration of Zn(II) ions were used to limit the quenching of the radical moieties as a means of controlling the modulation of the optical, magnetic, and electrochemical properties of MOFs.^[^ ^22^ ^]^ Tang et al. reported an exciting synthesis of radical MOFs where organic radical 2,4,6‐Tri(4‐pyridyl)‐1,3,5‐Triazine (TPT^•−^) was able to coordinate with

Na^+^ forming a framework.^[^ ^73^ ^]^ The guest molecule, THF could not be removed as it supported the stability of the MOFs. It is not commonly known for alkali metals to form MOFs. This suggests high reactivity of organic radicals with metals, although the 3D structure of the alkali framework was reported as unstable, and its lifetime was short. Even though there is a drawback of the short lifetime, this synthesis opens a window to synthesize stable radical MOFs using radical TPT^•−^ by designed reaction methods.

In summary, the integration of organic radicals into MOFs or the synthesis of radical MOFs has been achieved through various strategies. **Table** **1** demonstrates some examples of radical MOFs and organic radical inclusion MOF hybrids discussed above. The preparation approach, as well as corresponding properties or applications, are also presented in the table.

**Table 1:** Examples of radical MOFs and radical‐MOF inclusion compounds with their synthetic strategy, corresponding organic radical, and resulting properties/applications.

N^O^	MOFs	Synthetic strategy	Guest/ligand radicals	Application/properties	Reference
1	Zr‐PDI^•−^	Irradiation of MOF containing guest amines, inducing electron transfer	Perylenediimide ligand	Medical/photoconversion/ catalysis	[53]
2	RE_6_(*m*‐TTFTB)_2.5_(μ_3_‐OH)_8_ (H_2_O)_2_(HCOO)_2_] with TTF^•+^ moiety, RE: Tb, Dy, and Er	MOFs suspended in hexane containing iodine, promoting redox reaction	Tetrathiafulvalene‐tetrabenzoate ligand	Conductivity/photothermal properties	[39, 50]
	Zn‐MOF^•^	Postsynthetic visible light irradiation	[9,10‐bis(4‐pyridylethynyl)‐anthracene] derivative ligand	Photocatalysis	[68]
3	[Zr_6_(μ‐O)_4_(μ‐OH)_4_ (C_6_H_5_COO)_*x*_(OOCCH_3_)_4−*x*_ (BBI)_2_] radical	Postsynthetic irradiation of MOF containing acetone as electron donor	4,4′,4″,4‴‐(1,4‐phenylenebis(1H‐imidazole‐2,4,5‐triyl))‐ tetrabenzoic acid	Modulated spin states	[60]
4	[Zr_6_(μ_3_‐OH)_8_(OH)_8_](L‐OH)_2_	Postsynthesis light irradiation	2,2′‐dihydroxy‐1,1′‐ binaphthyl‐3,3′,6,6′‐tetrakis(4‐benzoic acid	Photocatalysis	[61]
5	[Co_3_(L)(N_3_)_4_]	Photoinduced irradiation	N,N′‐bis(3,5‐dicarbox‐ ylatobenzyl)‐4,4′‐bipyridinium	Magnetic	[1]
6	[Mn_2_(L)(N_3_)_2_(H_2_O)_2_]·3H_2_O	Dehydration at high temperature	N,N′‐bis(3,5‐dicarbox‐ ylatobenzyl)‐4,4′‐bipyridinium	Magnetic	[1]
7	UiO‐67 decorated by TEMPO	Solvothermal synthesis	TEMPO appended on biphenyl linker	Catalysis	[74]
8	[Cd_4_(L_1_)_2_(VOL_2_)_4_	Solvothermal synthesis	TEMPO‐substituted tricarboxylate as a radical linker	Catalysis	[7]
9	[Gd(bcbp•)(H_2_O)_2_]	In situ reduction of the ligand to form radical MOFs	1,1′‐bis(3,5‐dicarboxyphenyl)‐4,4′‐bipyridi‐nium	Photocatalysis	[51]
10	[Gd(bcbp•)(H_2_O)]	Removal of H_2_O upon heating to generate radical MOF	1,1′‐bis(3,5‐dicarboxyphenyl)‐4,4′‐bipyridi‐nium	Photocatalysis	[51]
11	Co(hfac)_2_	r.t synthesis using THF as solvent	4‐(2′‐pyridyl)‐1,2,3,5‐dithiadiazolyl as linker	–	[75]
12	{[Zn_4_(PTIA)(TPT)_2_(H_2_O)_2_]·7H_2_O)}	Postsynthetic UV light irradiation	piperazine‐N,N′‐ bis(2,4,6‐triazinyl‐3,5‐bis(N,N′‐iminodiacetic acid)), and 2,4,6‐ tri(4‐pyridyl)‐1,3,5‐triazine	Photochromic/inhibit cancer cell formation	[69]
13	Cd_3_(bipo^−•^)_4_(BDC)]_*n*_	Solvothermal	2,3′‐biimidazo[1,2‐α]pyridin‐2′‐one ligand	Magnetic and luminescent properties	[23]
14	Zn_2_(PHZ^•^)_2_(dabco)	Postsynthetic oxidation (AgSbF_6_: oxidizing agent)	‐5,10‐dihydrophenazine	Catalysis	[67]
15	[Ca_4_(μ_4_‐O)(L_2_^•−^)_2_(DMA^+^)_4_] or DGIST‐7	Solvothermal synthesis, where high temperature decomposes DMF to trigger electron transfer.	L= N,N′‐bis(3‐hydroxybenzoate)‐1,4,5,8‐naphthale‐ nediimide as radical linker	Catalysis	[54]

## 4 Features of Organic Radical Inclusion MOFs and Radical MOFs

The inclusion of organic radicals in MOFs offers the possibility of having an isoreticular network MOF, even though torsion and reduction in crystallinity may be observed in some structures.^[^ ^10^ ^]^ Changes in inclusion materials result from the host‐guest interactions. Inclusion systems behave differently depending on the size of pores or channels.^[^ ^12^ ^]^ The inclusion of radical PhCNSSN^•^ (PhCNSSN = phenyl dithiadiazolyl) into zeolite‐Y possessing different pore apertures was reported to influence the behavior of guest radicals in channels. Its large 12 Å pores are capable of accommodating the enthalpically preferred dimer, while the smaller pores dictate inclusion in the form of a paramagnetic monomer in zeolite‐Y.^[^ ^12^ ^]^ The dimers were formed through π*–π* interactions where similar interactions stabilized the radicals inside the cavities of the frameworks.^[^ ^59^ ^]^

Apart from inclusion, organic radicals are used as linkers to construct radical MOFs with an opportunity to have more accessible pores or channels.^[^ ^44^ ^]^ More importantly, some of those radical MOFs can be robust to withstand high temperatures up to 450 °C and maintain their structures,^[^ ^44^ ^]^ and even be stable in the presence of water or air.^[^ ^51^ ^]^ Such stability was also observed in [Gd(bcbp•)(H_2_O)_2_] framework (bcbp is 1,1′‐bis(3,5‐dicarboxyphenyl)‐4,4′‐bipyridinium where the ligands coordinating the frameworks are arranged in parallel orientation favoring radical‐radical and π–π interactions, which are responsible for stability.^[^ ^51^ ^]^

**Scheme 4:**
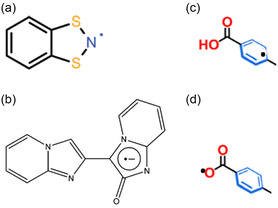
Radical feature on a) Nitrogen, b,c) aromatic ring, and d) oxygen.

The interactions between organic radicals as guest molecules or ligands and MOFs influence changes in the properties of the system. Hearns et al. used 1,2,3,5‐dithiadiazolyl (DTDA) radical derivative coordination with cobalt (II) to investigate the new magnetic properties^[^ ^75^ ^]^ They demonstrated that the radical ligand exhibited ferromagnetic coupling between its unpaired electron and those on a coordinated cobalt center, resulting in high spin hybrids with distinct spin‐ground states at lower temperatures. The unpaired electron characterizing the radical is always on a highly electronegative atom such as (a) N,^[^ ^49^, ^76^ ^]^ (b,c) in an aromatic ring,^[^ ^10^, ^23^ ^]^ and (d) O,^[^ ^44^, ^77^ ^]^ as depicted in **Scheme** **4**.

**Figure 9:**
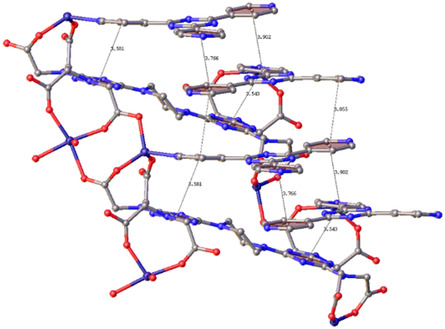
π–π interactions between PTIA and TPT ligands in [Zn_4_(PTIA)(TPT)_2_(H_2_O)_2_]·7H_2_O. Reprinted with permission.^[^ ^69^ ^]^ Copyright 2022, Royal Society of Chemistry.

Radical MOFs can be classified as anionic, cationic, or neutral due to the charge observed on the ligand, as the charge influences the rest of the MOF. Neutral radical MOFs are characterized by a balance of charges within the framework. Zhang et al.^[^ ^69^ ^]^ synthesized a photochromic radical MOF, {[Zn_4_(PTIA)(TPT)_2_(H_2_O)_2_]·7H_2_O)}((PTIA = piperazine‐N,N′‐bis(2,4,6‐triazinyl‐3,5‐bis(N,N′‐iminodiacetic acid)), TPT = 2,4,6–tri(4‐pyridyl)‐1,3,5‐triazine) where the ligands interact with each other via π–π stacking interactions, allowing the transfer of electrons from PTIA to TPT and creating opposite charges in either side of the ligands.^[^ ^69^ ^]^ Hence, the charges are balanced by the features of both ligands. **Figure** **9** displays a face‐to‐face interaction between PTIA and TPT. A similar mechanism of transfer was reported in 2,2′‐dihydroxy‐1,1′‐binaphthyl‐3,3′,6,6′‐tetrakis(4‐benzoate) linker of irradiated MOF.^[^ ^61^ ^]^ However, the process here is intramolecular proton transfer from photoinduction of MOF to convert the phenolic hydroxyl groups of the ligand into phenoxyl radicals.

Radical MOFs with anionic features were also prepared.^[^ ^23^, ^54^ ^]^ A framework is formed by an anion organic radical ligand, where the radical anionic moiety is maintained in the resulting MOFs.^[^ ^23^ ^]^ In this regard, anion radical ligand 2,3′‐biimidazo[1,2‐α] pyridin‐2′‐one (bipo^−•^) and co‐ligand, 1,4‐benzenedicarboxylate (BDC^2−^) were used to coordinate to Cd(II) and Fe(III) by a solvothermal reaction to form different radical anionic MOFs.^[^ ^23^ ^]^ Synthetic methods and metal sources showed that various crystal structures could be obtained from these mixed ligands. In addition, radical anionic MOFs are also prepared through postsynthetic modification based on induction by external stimuli such as redox processes.^[^ ^54^ ^]^

**Scheme 5:**
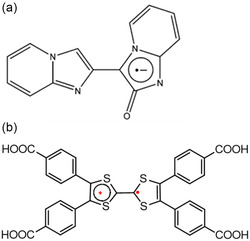
Examples of a) anionic ligands for radical MOFs. Reproduced with permission.^[^ ^23^ ^]^ Copyright 2012, Royal Society of Chemistry and b) cationic ligands for radical MOFs. Adapted with permission.^[^ ^39^ ^]^ Copyright 2021, John Wiley and Sons.

An example of radical cationic MOFs is the case whereby lanthanide nodes were coordinated to tetrathiafulvalene (TTF) forming frameworks where the latter were subjected to postsynthetic transformation. The powder MOF was placed in cyclohexane solution containing iodine and subsequently, partially oxidized TTF^•+^ was formed without altering the internal structure of the frameworks.^[^ ^50^ ^]^ Other radical cationic MOFs are reported to be generated via solvothermal in‐situ process as was observed in the synthesis of RE_6_(*m*‐TTFTB)_2.5_(μ_3_‐OH)_8_(H_2_O)_2_(HCOO)_2_]·1.5(NH_2_(CH_3_)_2_)·5DMF·8H_2_O where RE=Tb, Dy, and Er).^[^ ^39^ ^]^ The cationic charge is carried by TTF^•+^ ligand. These radical ionic MOFs always contain counterions in their channels or cavities of the frameworks to balance the charges. **Scheme** **5** presents examples of anionic and cationic organic radicals, respectively, for radical MOFs.

## 5 Effects of Inclusion of Organic Radical Moieties in MOFs on Physical and Chemical Properties

The inclusion of organic radical moieties in MOFs either as ligands in the fabrication of MOFs or as guests has been reported to enhance physicochemical properties of the resulting materials which is very important for applications in materials chemistry.^[^ ^27^, ^61^ ^]^ Once MOFs accommodate guest molecules or radical moiety, there are MOF–guest interactions that induce some transformation leading to hybrid materials with modified properties.^[^ ^10^, ^32^ ^]^ The changes include, for example, color change, stability vis‐a‐vis temperature or air/moisture, magnetism, optical properties, interatomic distances, reactivity, and sensing. The corresponding effects are discussed in the subsections below. These changes are key factors that can lead to novel applications. It can be noted that guest molecules behave differently in different hosts.^[^ ^59^ ^]^ This is because the interaction of guest molecules in one host will not be the same in another host, as the functionalities or backbones of both hosts are different. As a result, there would be an opportunity for varied applications for the same organic radical. Suitable surface area and size of pores or channel structures are very important factors for performance in adsorption or guest‐host contact.^[^ ^7^, ^51^, ^61^ ^]^ In some cases, the integration of organic radicals in pores can cause the confinement of the channels or pore structures leading to an increased catalytic activity or reactivity performance.^[^ ^7^, ^31^, ^68^, ^78^ ^]^

### 5.1 Reactivity of Organic Radicals and Organic Radical‐MOF Inclusion Compounds

The presence of an unpaired electron in organic radicals offers a highly reactive nature to the materials. This property influences some chemical changes in hybrid materials resulting from the combination of organic radicals with MOFs. There have been syntheses of different radical MOFs and organic radical inclusion in MOFs to develop properties such as magnetism, luminescence, and electrical conductivity.^[^ ^8^, ^10^, ^23^ ^]^ Upon inclusion in porous materials, the responsiveness of organic radicals may change, making it attractive to explore the inclusion compounds.^[^ ^12^, ^49^ ^]^

**Scheme 6:**
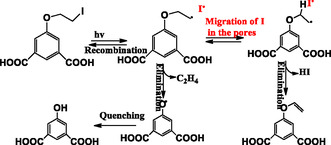
Transformation of **H**_**2**_ **lip** and **Znlip** after radical formation. Adapted with permission.^[^ ^65^ ^]^ Copyright 2021, Royal Society of Chemistry.

A change in reactivity of radical MOFs compared to the free organic radicals has been reported in some systems.^[^ ^65^ ^]^ **Scheme** **6** shows 5‐(2‐iodoethoxy)isophthalic acid (**H**_**2**_ **lip**) undergoing dissociation, forming alkyl and iodine radicals under photoirradiation. However, the alkyl radicals are short‐lived as they are still exposed to the iodine radicals. Therefore, they mostly react with iodine radicals to regenerate the acid. They can also be transformed to phenoxy radicals via intramolecular *β*‐cleavage eliminating ethylene followed by quenching to reach the most stable acidic form. On the other hand, the original ligand was coordinated to Zn^2+^ forming a MOF which further formed a radical **Znlip** MOF upon photoirradiation. Iodine radicals move along the channels away from the vicinity of the alkyl radicals and no quenching of the radical species is observed. Testing deactivation of radical species, oxygen was applied to both alkyl radical and radical MOF. The alkyl radical was quenched immediately while the radical MOF resisted for up to 300 min. The latter is the result of the confinement effects caused by the frameworks that reduce the exposure.^[^ ^67^ ^]^

In situ generated radical MOF was reported to have excellent photothermal conversion. Using *m*‐tetrathiafulvalene‐tetrabenzoate, (*m*‐TTFTB) trimer secondary building units (SBU) and hexanuclear Dy clusters, a radical MOF (Dy‐2D) was synthesized by solvothermal reaction.^[^ ^39^ ^]^ Dy‐2D was illuminated with simulated sunlight of 1–sun (0.1 W cm^−2^, 280–2500 nm), resulting in a temperature increase of 34.78C within 240 s. The structure of the radical MOF was characterized by intramolecular charge transfer, low thermal conductivity, and excellent stability which offer an excellent photothermal conversion. It was considered the first‐rate photothermal conversion performance under 1‐sun (0.1 W cm^−2^) relative to MOFs by the time it was reported.

**Figure 10:**
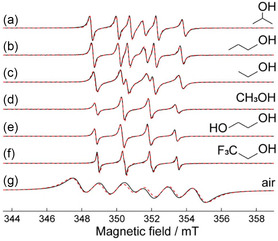
Identification of guest molecules using EPR spectra a) isopropanol, b) propan‐1‐ol, c) ethanol, d) methanol, e) ethylene glycol, and f) 2,2,2‐trifluoroethane, and g) air. Reprinted with permission.^[^ ^80^ ^]^ Copyright 2024, Royal Society of Chemistry.

Radical guest molecules in the channels of MOFs can selectively interact with other guest molecules. This was evidenced using a small concentration of stable nitroxides (TEMPO) in pores of ZIF‐8 (ZIF: zeolitic imidazolate framework), where reactive oxygen atoms interacting with radicals could be replaced by small organic molecules such as *p*‐, *m*‐, and *o*‐xylenes.^[^ ^55^, ^79^ ^]^ This switching was controlled by dipole−dipole interactions influenced by molecular mobility. As xylenes have different structures, their interactions with radical species inside ZIF‐8–TEMPO were identified by different shapes of EPR spectra. Note that *o*‐xylene could not penetrate into the channels, thus, the spectra remained as the ones of dried ZIF‐8‐TEMPO. Moreover, organic radicals were used to determine the environment (non‐polar and polar) of cavities in MOFs, structures, and the polarity of guest molecules. A determined quantity of stable *β*‐phosphorylated nitroxide radicals incorporated into ZIF‐8 allowed to explore different polarities of guest molecules such as water, small alkanes, and alcohols in the cavities.^[^ ^80^ ^]^ The obtained EPR spectra were specific to a certain molecule, **Figure** **10**. The shape or arrangement of EPR spectrum was displayed depending on the type of molecule. Therefore, a responsive tool for sensing molecules can be developed.

### 5.2 Stability and Catalytic Activity

Some compounds, including organic radicals are highly unstable and change into other undesirable forms easily. Organic radicals in channels of MOFs adopt the formation of dimers or trimers as building blocks that possess delocalized unpaired electrons, which result in chemical stability.^[^ ^11^, ^59^ ^]^ Polyphenols used as food additives were reported to become stable and ready to be used in their original forms after being incorporated into MOFs.^[^ ^47^ ^]^ These polyphenols as well as other organic radicals, once in the hollow of the frameworks, establish supramolecular interactions as mentioned above, giving rise to thermal and chemical stability compared to the pristine organic radicals. Organic radical anion of 7,7,8,8‐tetracyanoquinodimethane (TCNQ) was also investigated for stability of its free radical form, postsynthesis inclusion, and in‐situ inclusion in MOFs followed by exposure to water and high temperature.^[^ ^52^ ^]^ The lifetime in water of the free radical form was only 12 min while the lifetime in water for adsorbed radicals in ZIF‐8 was 2 days. In situ inclusion of radical into ZIF‐8 was performed using TCNQ in starting materials, and it was evident that the radicals coordinated with the metal ions.^[^ ^81^ ^]^ The resulting radical ZIF‐8 was also placed in water at 100 °C where the radical properties were maintained for 8 days. The stability of postsynthesis inclusion may be due to the limited access for air and reactive oxygen. While the higher stability of radical ZIF‐8 could be a result of both steric effects and new properties of the coordination phase.

While some radical MOFs can undergo crystal structure transformation to a more stable phase upon heating stimulus,^[^ ^51^ ^]^ there are also MOFs with organic radicals as linkers reported with high chemical stability. In this regard, *m‐*tetrathiafulvalene‐tetrabenzoate was used to synthesize RE_6_(*m‐*TTFTB)_2.5_(μ_3_–OH)_8_(H_2_O)_2_(HCOO)_2_]·1.5(NH_2_(CH_3_)_2_)·5DMF·8H_2_O, a 2D radical MOF, where this framework showed high stability in both organic and aqueous solutions.^[^ ^39^ ^]^ The extent of its stability was further proven in acid/base conditions (pH: 1‐12). Benefiting from the stabilization mechanism in the same radical MOF sunlight could irradiate the 2D radical MOF and its excitation stimulated intramolecular charge transfer, together with the lower thermal conductivity resulted in an outstanding photothermal conversion with an increase of 34.7 °C on 240 s of exposure.^[^ ^39^ ^]^ Moreover, perylenediimide ligand was also used to synthesize a MOF, which protected the radical anions from quenching by shielding effect in air and the radical anions remained undisturbed in the air for a minimum period of one month.^[^ ^53^ ^]^ This stability was considered as one of the factors that increased the higher near‐infrared photothermal conversion efficiency. Similar effects are reported on resistance to air observed in cycloparaphenylene radicals once incorporated in radical‐MOFs.^[^ ^27^ ^]^ This stability is a result of strong π–π interactions observed in radical frameworks.

Chemical and physical stability of organic radicals playing catalytic roles can be improved through their encapsulation in MOFs.^[^ ^82^ ^]^ The investigation on these effects was carried out on nanobiocatalyst (Ncat@ZIF‐8, CAT: catalase) which was prepared in situ by polymerization around CAT molecular radicals (noted as Ncat) followed by encapsulation of ZIF‐8 MOF via co‐precipitation method.^[^ ^82^ ^]^ Experimental analysis found that the catalytic activity of Ncat@ZIF‐8 (after encapsulation) was 3800 U g^−1^ protein compared to 4350 U g^−1^ protein of Ncat (before encapsulation), corresponding to about 87.35% of the original activity. Thermal stability was also improved when encapsulated one was found to retain 78.79% of its residual activity after incubation at 65 °C for 60 min compared to 48.95% before encapsulation. Moreover, repeated experimental investigations for catalytic activities for both CAT@ZIF‐8 and nCAT@ZIF‐8 were performed for comparison. The catalyst before encapsulation maintained only 67%, while after encapsulation it maintained 87.12%. Hence, it was confirmed that polymerization protected the enzyme activity in ZIF‐8 MOF shell while ZIF‐8 promoted the recyclability of the enzyme. The stability in this encapsulation was attributed to the presence of multipoint covalent linkage of polymer Ncat and MOF shells. Additionally, the protection from MOFs shells could reduce or limit heat transfer.

By integrating organic radicals in MOFs network, the heterogeneous catalytic activity and selectivity may become higher than the activity and selectivity of the homogeneous counterpart.^[^ ^7^ ^]^ In another study, a boosted photocatalytic reaction was achieved after incorporating Ru^II^(bpy)_3_ (where bpy: 2,2′–bipyridine) into a radical MOF, [Mn_3_(BINDI)_2_(H_2_O)Cl]·3Me_2_NH_2_(BINDI: N,N′‐bis(5‐isophthalate acid)naphthalene‐diimide, and NDI: naphthalene‐diimide).^[^ ^83^ ^]^ Before the inclusion, MOF and its homogeneous counterpart had afforded 7 and 56% of yield, respectively, of catalytic conversion of aerobic cross‐dehydrogenative coupling reaction of *N‐*phenyltetrahydroisoquinoline derivatives with phosphite esters into medicinal *α‐*aminoquinoline phosphonates. Note that BINDI was irradiated to form an organic radical ligand in the new MOF. After incorporation of Ru (II) complex into the radical MOF forming [Mn_6_(BINDI)_4_(H_2_O)_2_Cl_2_]·[Ru^II^(bpy)_3_]·4Me_2_NH_2_, a boosted photocatalytic conversion of 80% was achieved. The work suggested that the boosting of photocatalytic conversion might have been influenced by multiple intermolecular interactions (such as C—H··π and slipped π–π interactions) between NDI radicals and photoredox catalysts [Ru^II^(bpy)^3^].^[^ ^83^ ^]^

UiO–67–TEMPO MOFs were synthesized by varying the percentage of both unsubstituted linkers and TEMPO substituted linkers (as described in Section 3.2). Increasing the percentage of TEMPO substituted linkers resulted in reduced surface area but higher catalytic activity with limits.^[^ ^74^ ^]^ This was observed where UiO–67–TEMPO (38%) showed faster conversion for the aerobic oxidation of diphenylmethanol than both UiO–67–TEMPO (14%) and UiO–67–TEMPO (100%). However, the conversion in the latter was also faster than in UiO–67–TEMPO (14%). Effect of TEMPO for the best catalysts was evaluated in the catalytic aerobic oxidation of alcohols, **Table** **2**.^[^ ^74^ ^]^ Benzyl alcohol was converted into benzaldehyde using two catalysts for comparison in a range of solvents, including acetonitrile, acetone, toluene, and 1,2‐ dichloroethane (DCE). UiO–67–TEMPO (100%) synthesized MOFs showed larger pores but lower surface area, while UiO–67–TEMPO (38%) of TEMPO with higher surface area offered higher catalytic activities. The best catalyst was tested for recyclability and was reused three times. It remained crystalline but started to decompose to some extent after the first run. Moreover, the tested radical MOFs except UiO–67–TEMPO (100%), were stable in organic solvents but decomposed in water and highly acidic or basic solutions. UiO–67–TEMPO (100%) was more fragile, decomposing in highly acidic and basic solutions including Et3N, acetonitrile, and 1,4‐dioxane.

**Table 2:** catalytic activities by control TEMPO. Reprinted with permission.^[^ ^74^ ^]^ Copyright 2017, American Chemical Society.

Entry	Temperature	Solvent	yield of benzaldehyde [%]	
			UiO–67–TEMPO [*x*%]	
			*X* = 38	*X* = 100
1	25	dioxane	100	100
2	25	acetonitrile	100	91
3	25	toluene	38	56
4	25	water	100	26
5	25	1,2‐DCE	100	60
6	0	1,2‐DCE	48	4
7	05	1,2‐DCE	100	69
8	80	1,2‐DCE	100	80

MOF also containing phenazine radical ligand, Zn_2_(PHZ)_2_(dabco)·4DM showed high chemical and thermal stability useful for catalytic reactions.^[^ ^67^ ^]^ It was then used to catalyze aza‐Diels–Alder reaction. Not only was high conversion observed, but also the radical MOF could be recycled at least five times. It can be highlighted that radical species in MOFs catalyze certain reactions as well as the formation of other radicals.^[^ ^84^ ^]^

### 5.3 Magnetism and Electrical Conductivity

**Figure 11:**
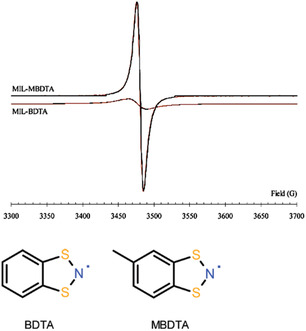
EPR spectra showing effects of symmetric and nonsymmetric radicals inclusion in MOFs. Adapted with permission.^[^ ^59^ ^]^ Copyright 2011, American Chemical Society.

The incorporation of organic radicals in MOFs has been used to control magnetic properties in MOFs.^[^ ^10^, ^49^, ^60^ ^]^ Also, magnetic properties of MOFs can be tuned by synthesizing MOFs using organic radicals or by transforming MOFs into radical MOFs on the application of stimuli such as light, UV, partial redox reaction,^[^ ^53^, ^73^ ^]^ or removal of guest molecules on heating followed by structural change.^[^ ^1^ ^]^ Dithiadiazolyl radical, PhCNSSN^•^ was incorporated into copper metallocycle [Cu_2_(L_1_)_2_Cl_4_] MOFs where L_1_ is the bidentate ligand 1,3‐bis(midazole‐1‐ylmethyl)‐2,4,6‐trimethylbenzene.^[^ ^11^ ^]^ The MOF itself is paramagnetic. While the inclusion led to a change and formed antiferromagnetic coupling between host Cu^2+^ ions and the guest PhCNSSN radicals, where the total number of spin centers increased. In addition, the stability of the organic radical became significantly enhanced. This is a result of the interaction between the host MOF and the organic radical that stimulates the change in the spin state of the organic radical. Note that the structure of radical guest molecules in MOFs plays an important role in the magnetic properties of the hybrid. A comparison can be made in the inclusion of dithiazolyl radicals MOFs. Herein, 5‐methylbenzo‐1,3,2‐dithiazolyl (MBDTA) and benzo‐1,3,2‐dithiazolyl (BDTA) radicals were included in MIL‐53 (MIL = Material of institute Lavoisier).^[^ ^59^ ^]^ **Figure** **11** illustrates that BDTA is symmetric, and its inclusion compound in magnetic field showed low intensity of EPR. This was a result of the interaction between guest radicals, which led to dimerization within the channels. On the other hand, nonsymmetric MBDTA in MILMBDTA hybrid, had shown a very high intensity of EPR in magnetic field.^[^ ^59^ ^]^ It can be suggested that the non‐symmetric structure bears steric effects that hinder the interaction between guest molecules, avoiding dimerization. This favors the optimization of host‐guest interactions, promoting an increase in paramagnetism in the sample.

On removal of water molecules from [Mn_2_(L)(N_3_)_2_(H_2_O)_2_]·3H_2_O (L = *N,N*′‐bis(3,5‐dicarboxylatobenzyl)‐4,4′‐bipyridinium, viologen ligand), magnetic χT value (where χ is magnetic susceptibility, T is temperature) was increased from 4.09 to 4.44 emu K mol^−1^ at 300 K.^[^ ^1^ ^]^ This was associated with radical generation from structural transformation that allowed electron transfer from electron‐rich (carboxylates or azide) to electron‐poor (viologen unit) moieties. The same radical MOF showed antiferromagnetic coupling between radicals, metal ions, or both, with the spin exchange between Mn(II) and the radical.^[^ ^1^ ^]^ Therefore, the effect of both increased temperature and removal of coordinated water molecules triggered radical formation and change in magnetic behaviors of the material. However, these changes only happen if factors such as distance between electron‐poor and electron‐rich sites, bridging angles, and torsion angles are arranged in a way that promotes these changes.

**Figure 12:**
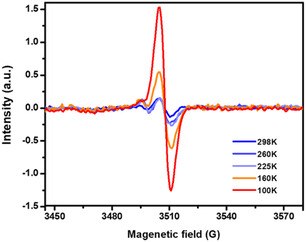
EPR signals relationship with temperature in **NU‐910**. Reprinted with permission.^[^ ^60^ ^]^ Copyright 2022, American Chemical Society.

Temperature change has also been probed for its effect on magnetic properties of radical compounds. **Figure** **12** shows EPR spectrum of [Zr_6_(μ–O)_4_(μ–OH)_4_(C_6_H_5_COO)x(OOCCH_3_)_4−*x*_ (BBI)_2_], **NU‐910** where BBI is 4,4′,4″,4‴‐(1,4‐phenylenebis(1H‐imidazole‐2,4,5‐triyl))‐tetrabenzoate linker, the intensity decreased once the temperature is increased.^[^ ^60^ ^]^ At a temperature above 225 K, a very weak signal was detected by EPR, which was the result of the formation of dimer radicals and a diamagnetic singlet state, the radical π–dimer. The main cause of the reduction of EPR signals at higher temperature was attributed to shortened interlinker distance (3.19 Å) in MOFs, which subsequently caused strong intermolecular interaction between adjacent radical linkers. While at a temperature of 160 K and below, the interlinker distance (3.63 Å) is longer, maintaining paramagnetic organic radical isolated. On the other hand, there are radical compounds that do not show a change in EPR signals by increasing or decreasing the temperature. Moreover, variation of temperature under vacuum does not show an important change as there is no oxygen to generate more radical species on organic ligands. Chen et al.^[^ ^60^ ^]^ found that this is because the organic radicals remain isolated without the formation of dimers. This behavior may also be attributed to rigid frameworks, which resist stimulus, compared to flexible and breathing frameworks, which undergo contraction or expansion.^[^ ^35^ ^]^ Moreover, the magnitude of magnetic susceptibility may also be influenced by the nature of the metal centre.^[^ ^8^, ^39^ ^]^ This was observed in MOFs such as Yb_6_(TTFTB)_5_ and Lu_6_(TTFTB)_5_ radical MOFs, (TTFTB: tetrathiafulvalene tetrabenzoate), where Yb_6_(TTFTB)_5_ was characterized by higher magnetic susceptibility compared to Lu_6_(TTFTB)_5._^[^ ^8^ ^]^ This was explained by the fact that magnetic susceptibility is a result of both paramagnetic components from metal centers (χ_P_) and temperature‐independent paramagnetism (TIP) contribution from ligand spins (χ_TIP_).

As discussed above, the presence of radicals in MOFs is very important for applications as their interaction within the frameworks enables or enhances important physical and chemical properties, among which is electrical conductivity.^[^ ^8^ ^]^ Electrical conductivity after radical inclusion in MOFs or electrical conductivity of radical MOFs is also expected as a strong resonance in a continuous‐wave EPR spectra, as it was reported for TTFTB^•+^ radical.^[^ ^8^ ^]^ High electrical conductivity is however supported by orbital overlap between dimers or trimers, allowing the charge transport. The ability of the ligands to allow free charge carriers due to their partial oxidation makes such MOFs electrically conductive.^[^ ^8^, ^85^ ^]^ Similar creation of conductivity was reported in Ni(II) Tetraaza[14]annulene‐linked MOF.^[^ ^85^ ^]^ Enhanced electrical conductivity in MOFs was also reported on the incorporation of TCNQ radicals into [Cu_3_(BTC)_2_] (BTC = 1,3,5‐benzenetricarboxylate). In this incorporation, TCNQ radical species are coordinated to the Cu^2+^ centers with the generation of charge carriers, resulting in higher conductivity.^[^ ^81^ ^]^ It was reported that radical guest molecules that coordinate to the metal centers induce higher electric conductivity than those that do not coordinate.^[^ ^86^ ^]^ Ligands such as pyrizine, 2,5‐dihydroxy‐1,4‐benzoquinone, and its derivatives,^[^ ^87^ ^]^ and triphenylmethyl carboxylate radical derivatives^[^ ^22^ ^]^ in MOFs undergo redox reactions to generate appreciable magnetic and conducting properties. Computational studies on benzoquinoid frameworks were conducted to demonstrate the reduction of the benzoquinone ligand (L^2−^, *S* = 0) to a radical ligand (L^3−•^ with *S* = 1/2). The latter showed the antiferromagnetic coupling with Fe and Mn metal ions, and their π−d overlap resulted in a greater delocalization charge, which is a factor for increased electric conductivity.^[^ ^87^ ^]^ Besides, higher conductivity was obtained for the Fe compound compared to the Mn one due to higher magnification of electron delocalization for the reduced Fe compound. Hence, it is likely that the conductivity in MOFs will vary depending on the metal center and their respective π−d overlaps. For MOFs possessing interacting layers, their electrical conductivities can be controlled by the introduction of bulky side groups between adjacent ligands.^[^ ^88^ ^]^ This was reported in [Ni_3_(HATI_X)_2_ MOF (HATI: π‐conjugated 2,3,7,8,12,13‐hexaiminotriindole ligand), where the functionalization of HATI using n‐propyl, and isopropyl groups weakened the interaction between layers, resulting in increased spin density while the conductivity and spin relaxation time are reduced.^[^ ^88^ ^]^ It is evident that reducing bulky groups will reverse the properties. Hence, the control of spin state would be an advantage for the application in superconducting circuits.^[^ ^89^ ^]^

### 5.4 Optical Properties

Some MOFs, as well as organic radicals were reported to exhibit optical properties, thus making them important for use in materials for solar cells, bioimaging, in the buffer layer between electrodes, and optoelectronic technologies.^[^ ^50^, ^83^, ^90^, ^91^ ^]^ It is known that the optical properties of materials indicate information regarding the characteristic bandgap, and occupied and unoccupied parts of the electronic structure.^[^ ^90^ ^]^ Bennett et al.^[^ ^92^ ^]^ concluded on the possibility of tuning porous metal–organic networks into optically active materials through the inclusion of chiral sorbate molecules. Organic radicals are thus suitable for such inclusion to achieve the optical properties. Monoradicals contain singly occupied molecular orbitals (SOMO) with abilities of charge transfer and storage by transferring electrons to or from the surroundings. These materials, referred to as open‐shell organic radicals, are characterized by low‐lying excited states which generate attractive optical properties.^[^ ^93^ ^]^ Moreover, they overcome theoretical limit of fluorescent organic light emitting materials through their unique luminescent emission from spin doublet excited state to doublet ground state.

The optical properties in MOFs are influenced by hydrogen bonding, lone pair−π interactions, π–π interactions, electrostatic effects, coordination, framework arrangements, and electron transfer.^[^ ^50^, ^62^, ^94^ ^]^ Moreover, optical properties are a result of partial degree of charge transfer between host framework (donor) and guest molecule (acceptor).^[^ ^81^ ^]^ According to the information in the sections above, it can be seen that a MOF containing organic radical is characterized by donor: azide−carboxylates moieties susceptible to transfer electrons to acceptor: viologen, and this gives rise to optical properties.^[^ ^1^ ^]^ In addition, the more supramolecular interactions are established between host‐guest, the more optical properties can be tuned. Hence, the radical MOFs or inclusion of organic radicals into MOFs may lead to the development of advanced optical materials.

### 5.5 Sensing Agent

**Figure 13:**
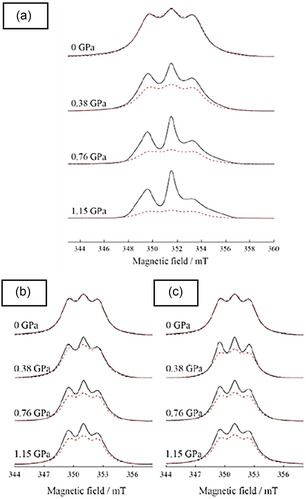
Integrated room‐temperature EPR spectra characterizing the level of amorphization of ZIF‐8–TEMPO with applied pressures of 0.38, 0.76 and 1.15 GPa. a) Without guest molecules (experimental spectra are shown in solid black, simulation of intact fractions are in dashed red), b) with isopropanol (as shock absorber), and c) with toluene (as shock absorber). Experimental spectra are shown in solid black and simulations of bare TEMPO@ZIF‐8 are in dashed red. Reprinted with permission.^[^ ^95^ ^]^ Copyright 2020, American Chemical Society.

Organic radicals can act as agents to monitor external stimuli‐induced processes in MOFs.^[^ ^55^, ^56^, ^95^ ^]^ This was illustrated by the use of stable nitroxide radicals in ZIF‐8 (ZIF‐8–TEMPO), where the intensity of integrated EPR spectra at room temperature was inversely proportional to the level of applied external pressure (0, 0.38, 0.76, and 1.15 GPa) on ZIF‐8–TEMPO containing shock absorber‐like molecules or not.^[^ ^95^ ^]^ **Figure** **13**a shows that ZIF–8–TEMPO without applied pressure had higher intensity (of integrated EPR spectra) than the ones affected by the pressures. The contribution to the spectra originates from intact cavities of MOF, which offer a high rotation of TEMPO, while the damaged cavities offer limited rotation of TEMPO. This was used to monitor the level of amorphization in ZIF‐8–TEMPO, containing shock absorber‐like molecules such as toluene and isopropanol. The shock absorber‐like molecules were used to counteract the amorphization of ZIF‐8. Figure 13b,c show integrated EPR spectra when ZIF‐8–TEMPO containing toluene or isopropanol guest molecules was subjected to a range of external pressures such as 0, 0.38, 0.76, and 1.15 GPa. Note that the spectra result from the activated ZIF–8–TEMPO after removing guest molecules.

The contribution to the EPR spectra of the undamaged or intact fraction of ZIF–8–TEMPO without shock absorber‐like molecules denoted as int, was obviously 100% prior to pressure application, and it decreased to int = 70% after 0.38 GPa, to int = 50% after 0.76 GPa, and to int = 30% after 1.15 GPa pressures were applied to the MOF. Whereas such pressures were applied on ZIF–8–TEMPO with shock absorber‐like molecules and a higher contribution of the intact fraction was observed e.g., int = 90, 80, and 80%, respectively, for ZIF‐8–TEMPO with isopropanol. Similarly, intact fractions, int = 90, 80, and 80%, respectively, were observed for ZIF‐8–TEMPO with toluene (as a shock absorber). Hence, the efficiency of counteracting the amorphization was determined. As the external pressure is applied, some MOF fractions are destroyed, and an amount of organic radicals are evacuated from the pores of the destroyed fractions. EPR intensity is therefore relative to the remaining radical molecules in intact fractions. In general, the gradual decrease of intensity is relative to the degree of applied pressure to the framework.

In addition to the characterization of the stability of the frameworks, Section 5.1 discussed the use of nitroxide radicals for molecular recognition of guest molecules. Organic radicals can also be used to quantify the concentration of molecules stored or released from the cavities of MOFs in drug release conditions.^[^ ^96^ ^]^ This was easily indicated by the gradual change of the shape (broad or narrow) of the EPR spectra. Moreover, the embedded radical moieties generated from a spontaneous oxidation of tritopic ligand 2,3,6,7,10,11‐hexahydroxytriphenylene (HHTP) in MgHOTP MOF were used to quantify the Li^+^ concentration in a range of (5^×3–10^−2 mol L^−1^ using EPR spectroscopy.^[^ ^97^ ^]^ It was demonstrated that increasing the concentration of Li^+^ reduced the spin‐lattice relaxation time (T1) and phase memory time (Tm), which indicates a stronger radical‐Li^+^ hyperfine interaction. To this end, the inclusion of organic radicals has the potential to sense some new molecules and pressure stimuli. However, the investigated MOFs presented limitations for the polarity of the solution and selectivity respectively.

### 5.6 Other Applications

**Figure 14:**
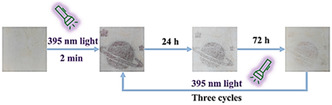
Photograph of the content printed on one coated paper with a planet motif, the printing of the content after 24 h, the printing of the content after 72 h, and after printing for the 3rd cycle. Reproduced with permission.^[^ ^100^ ^]^ Copyright 2022, Chemistry ‐ An Asian Journal.

The inclusion of organic radicals in MOFs is also investigated for potential in cancer treatments. One of the candidates is Zr‐MOF carrying 2,2′‐azobis[2‐(2‐imidazolin‐2‐yl)‐propane]dihydrochloride (AIPH) radical, Zr‐MOF@AIPH.^[^ ^98^ ^]^ Upon application of ultrasound irradiation, AIPH embedded in Zr‐MOF can form an alkyl radical. The latter can further produce reactive oxygen species (ROS), which proved to kill tumor cells. The combination of MOFs and AIPH may increase efficacy through charge transfer from AIPH to MOFs, even though this mechanism is not clear yet.^[^ ^99^ ^]^ While AIPH was reported to be chemically unstable in vivo, Zr‐MOF@AIPH showed appreciable stability both in vivo and in vitro, and in various solutions.^[^ ^99^ ^]^ This would attract the attention of various researchers to use and develop similar materials for anti‐cancer tools. Organic radicals in MOFs or radical MOFs can be used in inkless printing or information security applications. This was investigated where N,N′‐bis(3‐ pyridylcarbonylhydrazine)‐1,4,5,8‐naphthalene diimide (NDI) linker was used with Cd metal center to construct a radical MOF, {[Cd_3_(NDI)_3_(IA)_3_]⋅3H_2_O}n where IA is isophthalic acid.^[^ ^100^ ^]^ Acetonitrile was used to adjust electron transfer within the framework by increasing interfacial interaction between electron donors and electron acceptors. This increased charge transfer results in improved photochromic process. The obtained MOF material was printed on a cellulose filter paper in the form of a planet. It was then irradiated by purple light for two minutes, displaying planet picture information (**Figure** **14**). Once the light was removed, the information stayed for three days and could persist for up to seven days before fading away. This process could be repeated, proving reusability. The development of this technology is with no doubt environmentally friendly.

The presence of radical moieties in MOF frameworks and pathways for electron or charge transfer makes the frameworks more sensitive. They can also provide electrostatic interaction to improve the adsorption capacity for CO_2_ and its selectivity. In this regard, electrostatic interactions were investigated for MIL‐101 functionalized with poly(ionic liquid)s.^[^ ^101^ ^]^ The interactions aided the preferential transport of CO_2_ while repelling CH_4_. Hence, acted as a tool for CO_2_/CH_4_ separation. Therefore, radical moieties in MOFs can be explored for improved CO_2_ adsorption.

## 6 Conclusion and Outlook

Incorporation of organic radicals in MOFs is very important for the development of novel materials with potentially new properties. Organic radicals can also be used as ligands in the synthesis of radical MOFs and the latter can be considered as an alternative to the inclusion compounds. As MOFs possess pores or channels, they can accommodate different organic radicals, generating new properties. Hence, unstable organic radicals can attain stability through inclusion in MOFs. This review has discussed different inclusion methods such as diffusion and in situ synthesis of organic radicals in MOFs, where the method mostly depends on the nature or volatility of organic radicals. As a result, properties such as magnetic properties, electrical conductivity, and thermal stability have been improved. Thus, the combination of organic radicals and MOFs in solid‐state leads to new chemistry materials. The inclusion results in supramolecular interactions between guest and host which may not alter the host structure. This is an essential property not found in many inclusion compounds.

Radical MOFs can be prepared in situ from organic radicals and metal salt, doping, or the use of guest molecules able to induce electron transfer. Postsynthesis methods using irradiation, heating, or electron donor compounds are also used. One factor that determines the creation of a radical MOF is its structure. In isoreticular MOFs, radical formation may be possible in one MOF but not in another due to a different metal ion. In addition, the formation of radical MOFs depends on the nature of the ligand that allows electron‐transfer in acceptor‐donor moieties. Radical MOFs can be classified into neutral, anionic, and cationic radical MOFs. They also show changes in reactivity compared to their counterpart and improved properties such as catalytic activity, magnetic properties, electrical conductivity, and optical properties. These properties are controlled by electron transfer, charge transfer and π–conjugation molecular systems. Therefore, the production of radical MOFs or organic radical‐MOF inclusion compounds is attractive in applications such as catalysis, solar cells, semiconductors, and bioimaging devices. Among the practical applications of radical MOFs or radical‐MOF inclusion hybrids are also the treatment of cancer, printing of information on paper or imaging. More research efforts should be devoted to the synthesis of these materials. The potential of organic radicals inclusion in MOFs to sense applied pressure or chemical environments in MOFs could be investigated more for efficient conductivity and catalysis. It is noteworthy that after organic radical inclusion in MOFs, the accessibility to channels or pores can be limited or even occluded. Consequently, this may hinder some reactions that depend on adsorption including catalysis. Therefore, more research on radical MOFs can be more useful than organic radical‐MOF inclusion compounds for the access of guest molecules to the channels. Radical MOFs showed that they can allow adjustment for electrical conductivity, which is attractive for quantum information science. More studies are needed, as this is still in its infancy.

## Conflict of Interest

The authors declare no conflict of interest.
